# Extent, trends, and determinants of controller/reliever balance in mild asthma: a 14-year population-based study

**DOI:** 10.1186/s12931-019-1007-0

**Published:** 2019-02-28

**Authors:** Amir Khakban, J. Mark FitzGerald, Hamid Tavakoli, Larry Lynd, Solmaz Ehteshami-Afshar, Mohsen Sadatsafavi

**Affiliations:** 1 Collaboration for Outcomes Research and Evaluation, Faculty of Pharmaceutical Sciences, the University of British Columbia, Vancouver, Canada; 2 Division of Respiratory Medicine and Institute for Heart and Lung Health, Vancouver General Hospital, the University of British Columbia, Vancouver, Canada; 3 Respiratory Evaluation Sciences Program, Faculty of Pharmaceutical Sciences, the University of British Columbia, Vancouver, Canada; 4 Center for Health Evaluation and Outcome Sciences, Vancouver, Canada

**Keywords:** Asthma, Mild asthma, Controller medication, Reliever medication, Trend

## Abstract

**Background:**

The majority of patients with asthma have the mild form of the disease. Whether mild asthma patients receive appropriate asthma medications has not received much attention in the literature. We examined the trends in indicators of controller/reliever balance.

**Methods:**

Using administrative health databases of British Columbia, Canada (2000 to 2013), we created a population-based cohort of adolescents/adults with mild asthma using validated case definition algorithms. Each patient-year of follow-up was assessed based on two markers of inappropriate medication prescription: whether the ratio of controller medications (inhaled corticosteroids [ICS] and leukotriene receptor antagonists [LTRA]) to total asthma-related prescriptions was low (cut-off 0.5 according to previous validation studies), and whether short-acting beta agonists (SABA) were prescribed inappropriately according to previously published criteria that considers SABA in relation to ICS prescriptions. Generalized linear models were used to evaluate trends and to examine the association between patient-, disease-, and healthcare-related factors and medication use.

**Results:**

The final cohort consisted of 195,941 mild asthma patients (59.5% female; mean age at entry 29.6 years) contributing 1.83 million patient-years. In 48.8% of patient-years, controller medications were suboptimally prescribed, while in 7.2%, SABAs were inappropriately prescribed. There was a modest year-over-year decline in inappropriate SABA prescription (relative change − 1.3%/year, *P* < 0.001) and controller-to-total-medications (relative change − 0.5%/year, *P* < 0.001). Among the studied factors, the indices of type and quality of healthcare (namely respirologist consultation and receiving pulmonary function test) had the strongest associations with improvement in controller/reliever balance.

**Conclusions:**

Large number of mild asthma patients continue to be exposed to suboptimal combinations of asthma medications, and it appears there are modifiable factors associated with such phenomenon.

**Electronic supplementary material:**

The online version of this article (10.1186/s12931-019-1007-0) contains supplementary material, which is available to authorized users.

## Background

Asthma is one of the most common chronic diseases worldwide. While it cannot be cured, achieving clinical and symptomatic control can substantially reduce the burden of asthma. Evidence shows that achieving control is an attainable goal in the majority of patients, especially those with mild disease [1]. The cornerstone of pharmacological asthma management is controller medications with anti-inflammatory effects, namely inhaled corticosteroids (ICS), as well as leukotriene receptor antagonists (LTRA) [2]. On the other hand, many patients perceive immediate symptom relief through the use of reliever (or rescue) medications, such as inhaled short-acting beta-agonists (SABA), which cause rapid resolution of symptoms through the temporary relaxation of airway smooth muscles. However, reliever medications do not tackle the core underlying inflammatory mechanisms of asthma. Patients who mainly rely on reliever medications are at increased risk of periods of intensified disease activity commonly known as exacerbations or attacks. One postulated mechanism for such an increased risk is that through symptomatic relief, reliever medications facilitate inhalation of exacerbation triggers which, in the absence of anti-inflammatory therapy, make patients particularly vulnerable to severe and potentially fatal attacks [3–5]. Indeed, landmark studies have demonstrated that monotherapy with SABAs is associated with an increased risk of severe exacerbations and mortality [6, 7].

While adverse outcomes such as severe exacerbations and mortality mainly affect patients with severe asthma, the population-level consequences of inappropriate use of reliever medications among mild asthma patients might be larger given the vastly higher prevalence of mild asthma. Indeed, recent evidence suggests that controller/reliever balance in mild asthma is directly associated with risk of poor symptom control and exacerbations. The SYGMA trial convincingly demonstrated that as-needed budesonide (ICS) plus formoterol (a rapid-onset long-acting beta-agonist [LABA]) provided superior asthma-symptom control to as-needed terbutaline (SABA) in terms of weeks with well-controlled asthma, while budesonide maintenance therapy was superior to both regimens. Exacerbation rates with the two ICS-containing regimens were similar and were significantly lower than the rate with as-needed SABA therapy [8]. In a post hoc analysis of data from more than 7000 mild asthma patients from the multinational Steroid Treatment As Regular Therapy (START) study, Reddel et al. demonstrated that even in the mildest form of asthma (symptoms 0–1 days per week), maintenance ICS therapy reduced the combined risk of severe asthma-related outcomes (defined as any of asthma-related hospital admission, emergency treatment, or death). This benefit was consistently observed for both persistent and intermittent mild asthma [9]. In a previous work, we have demonstrated the adverse consequences associated with such inappropriate use in a cohort of largely mild asthma patients [10].

Given such recent findings, it is important to understand the current status of medication use in mild asthma. Such findings can enable estimating the population-level burden of mild asthma that is potentially preventable through the promotion of evidence-informed treatments. Such information is crucial to evaluate the return-on-investment of programs and policies towards improving the management of mild asthma. Accordingly, the purpose of the present study was to document the extent, trends, and predictors of the balance between controller and reliever medication use in mild asthma, using a population-based longitudinal database.

## Methods

### Study population

We used administrative health databases of all legal residents of British Columbia (BC), from 1997 to 2014. BC is a Canadian province with a population of 4.65 M (as of 2016, [11]) with a universal healthcare system, whose administrative needs have resulted in the creation of centralized datasets that capture all resource use records of legal residents in the province. We obtained the linked births and deaths data [12], records of inpatient care episodes [13], outpatient services use [14], and medication dispensation [15]. We also had access to census data that provided estimates of neighborhood income as a proxy for socio-economic status [16]. This study was approved by the University of British Columbia’s Human Ethics Board (application H15–00062). All inferences, opinions, and conclusions drawn in this research are those of the authors and do not reflect the opinions or policies of the Data Steward(s).

From these datasets, we created a cohort of adolescent/adult asthma patients using a validated case definition of asthma [17]. According to this definition, an individual is considered as having diagnosed asthma if they satisfy one or more of the following three criteria in any rolling 12-month period: 1) filled prescriptions on three different dates for at least three asthma-related medications, or 2) had two outpatient asthma-related visits on different dates, or 3) had at least one hospitalization with the main discharge code of asthma diagnosis (however, while hospital-based component is part of this validated algorithm, further restriction of the sample to mild asthma cases removed the subgroup of patients who entered the main asthma cohort through an instance of asthma-related hospital admission). We used international classification of disease (ICD), 9th revision code of 493.xx and ICD, 10th revision codes of J45-J46 for identifying asthma-specific healthcare resource use. Asthma-related medications were determined based on expert opinion (list provided in Additional file 1: Table S1). The above-mentioned criteria were applicable for the period in which the individual was between ages of 14 and 45. The upper age limit was chosen to reduce the risk of misclassification due to inclusion of patients with chronic obstructive pulmonary disease. However, once patients were identified during this period, they could remain in the cohort regardless of their age (resulting in the upper age limit in the cohort being 59 years). We did not include the first three years of data (1997–1999) in the analyses to allow sufficient time for patients with asthma to be identified. Also, we excluded the last year of data (2014), as the 12-month rolling window for asthma definition would result in under-representation of asthma patients in this year.

We considered the first date of any asthma-related resource use after the patient’s 14th birthday as the index date, marking the beginning of follow-up. Follow-up was terminated at the earliest date of last resource use of any type, death, or the administrative end of study (Dec. 31th, 2014). The follow-up time for each patient was divided, starting from the index date, into juxtaposing 12-month intervals (henceforth referred to as ‘periods’) during which study outcomes were measured.

### Assessing asthma severity

We used a validated algorithm, developed using Canadian administrative databases [18], to classify each period into mild or non-mild asthma. This algorithm classifies a patient-year as mild asthma according to the combination of absence of exacerbation markers (e.g., emergency department visits, oral corticosteroid use, or hospital admissions) as well as asthma medication patterns, and has been validated, using chart review, against the Canadian Asthma Consensus Guidelines [18].

### Outcomes

The two co-primary outcomes of interest were suboptimal prescription of controller medications and inappropriate prescription of SABA, both defined during each period for every patient using previously used and validated metrics. Suboptimal controller prescription was defined as a period in which the ratio of total dose of ICS (beclomethasone equivalent) and LTRA (all considered having equal potency) to total dose of all asthma-related medications was less than 50% [19]. This is a validated metric of controller/reliever balance with an established association with asthma outcomes [19]. This metric in its original for does not consider different potencies of inhaled medications. A previous study has demonstrated that both dose-adjusted and unadjusted metrics of ICS use were predictive of adverse asthma outcomes [20]. However, we decided to incorporate such dose-equivalence information into calculations due to the perceived importance of ICS dosing in mild asthma give the recent evidence [9]. Inappropriate prescription of SABA was defined as a period in which a patient had filled prescriptions corresponding to greater than two puffs of SABA per week without any concomitant ICS prescription, or nine or more canisters of SABAs per year and no more than an average of 100 μg (beclomethasone equivalent) per day of ICS [21]. This metric has been used previously and has been shown to be associated with adverse outcomes and increased costs. The above-mentioned outcomes were defined only in periods in which the patient filled a prescription for at least one asthma-related medication. The asthma was considered dormant in the other periods and such periods were not included in the subsequent analyses. All inhaled medications were dose-adjusted according to the established dose-equivalence information (Additional file 1: Table S1).

### Statistical analysis

The trends of the two outcomes were quantified over three time axes: calendar year, time since mild asthma diagnosis, and age. Each period was assigned to each calendar year depending on the date at the beginning of the period; similarly, the age of the patient was assessed at the beginning of each period. Only periods of mild asthma were included in the analyses. For the trends over time since the incidence of mild asthma, a subgroup of incident mild asthma was created consisting of patients whose first year of asthma was classified as mild and who were present for at least five years in the data before their asthma diagnosis. We tested the trends in outcomes using Generalized Linear Models (GLM) with Poisson distribution and logarithmic link function, in which the number of periods with suboptimal controller prescription or inappropriate SABA prescription was the dependent variable, and the logarithm of the total number of periods was the offset variable.

For associating factors with suboptimal controller prescription and inappropriate SABA prescription, we fitted GLM models with binomial distribution and logit link function (equivalent of conventional logistic regression but accommodating the clustered nature of data [multiple periods for each patient]). The dependent variables for this analysis were suboptimal prescription of controllers and inappropriate prescription of SABAs in the current period, and the independent variables were a series of covariates in the same or in the immediate preceding period. These were variables pertaining to socio-demographic characteristics, asthma-related treatments, comorbidity burden (including the Charlson comorbidity index [22]), and variables representing type (specialist versus primary care) and continuity of care. The latter was measured using the Bice-Boxeman index for each patient-year [23]. This index varies between 0 and 1, with zero meaning that an individual’s physician visits were all to different physicians during the year, and 1 meaning that the individual only consulted with the same physician during the year.

*P*-values (P) were considered significant at the 0.05 level (two-tailed). SAS Enterprise Guide (version 7.3, SAS Institute, Cary, NC, USA) was used for data linkage and preparation steps and for statistical analyses.

## Results

A total of 201,289 patients met the case definition of asthma. Among these, 195,941 (mean age at entry 29.6 years, 59.5% female) had at least one period categorized as mild asthma and thus were included in the analytical dataset (Table 1). The incident cohort of asthma included 88,110 individuals.

**Table 1:** Follow-up statistics and characteristics of study population

Variable	Cohort
Total sample	195,941
Patient years	1,827,150
Female; N (%)	114,673 (59.5%)
Age at index date; Mean (SD)	29.6 (9.55)
Socio-economic status; N (%)	
Quintile 1	19,014 (9.7%)
Quintile 2	26,332 (13.5%)
Quintile 3	35,420 (18.2%)
Quintile 4	47,858 (24.6%)
Quintile 5	64,326 (33%)
Unknown	1824 (0.9%)
Follow up years; Mean (SD)	10.7 (4.95)
Ratio of ICS to total asthma medications; Mean (SD)	0.44 (0.400)
Suboptimal use of ICS (< 50%)	373,911 (48,8%)
Inappropriate use of SABAs; N (%)	55,414 (7.2%)

*ICS* inhaled corticosteroids, *SABA* Short-acting beta agonist, *SD* Standard deviation

### Extent of suboptimal controller and inappropriate SABA prescriptions

The average ratio of total dose of controllers to total dose of all asthma-related medications was 0.44 (SD 0.4). In 48.8% of periods, controllers were prescribed suboptimally (ratio < 0.5), and in 7.2% of periods SABAs were prescribed inappropriately.

### Trends over calendar time

**Fig. 1:**
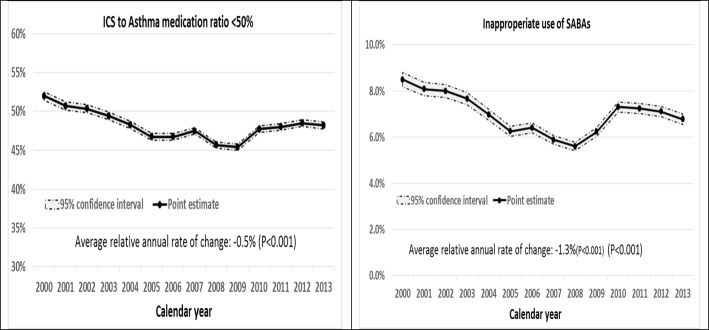
Trend of controller prescription to total medications (left panel) and inappropriate use of SABAs (right panel) over calendar year

Trend of controller-to-total-asthma medications ratio showed a slight drop from 52 to 48.2% from 2000 to 2013 (Fig. 1- left panel). The annual trend was slightly downward, with a relative decline of 0.5% per year (*P* < 0.001). Inappropriate prescription of SABAs decreased from 8.5 to 6.8% during the same period. Aside from an upward trend in inappropriate prescription of SABAs in the 2008–2010 period, the overall trend was decreasing, with an annual relative decline of 1.3% (*P* < 0.001) (Fig. 1**-** right panel).

### Trends over age

**Fig. 2:**
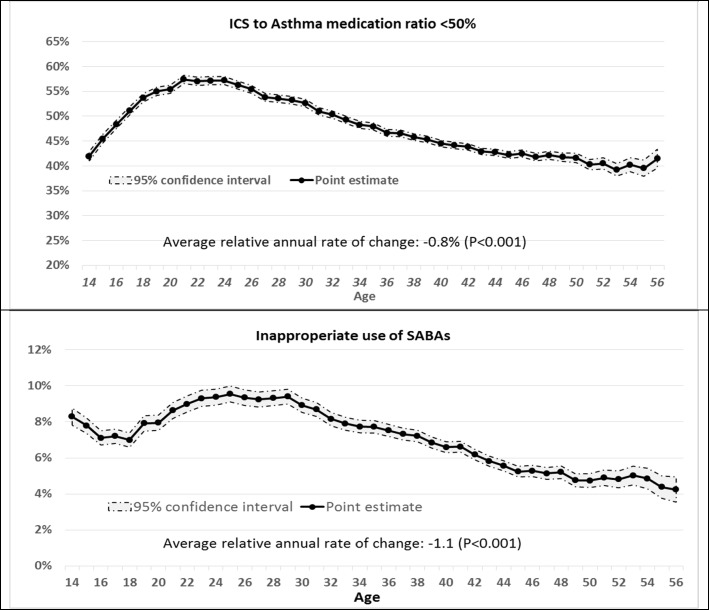
Trend of controller prescription to total medications (top panel) and inappropriate use of SABAs (bottom panel) over age of mild asthma patients

Trends are demonstrated in Fig. 2. The proportion of patients with suboptimal prescription of controllers generally increased over young age groups, from 43% in 14 years to 57% in 24 years of age. This proportion decreased to 44% by age 40, and remained mostly constant afterwards. The overall trend was consistent with a relative decrease of 0.8% per year of age (*P* < 0.001). Inappropriate prescription of SABAs declined from 8.3% at age 14 to 7.0% at age 18. This ratio increased to 9.4% by age 24–29 and then gradually declined, plateauing around 5.5%. Overall, the trend was consistent with a relative decline of 1.1% per year of age (*P* < 0.001).

### Trends over time course of asthma

**Fig. 3:**
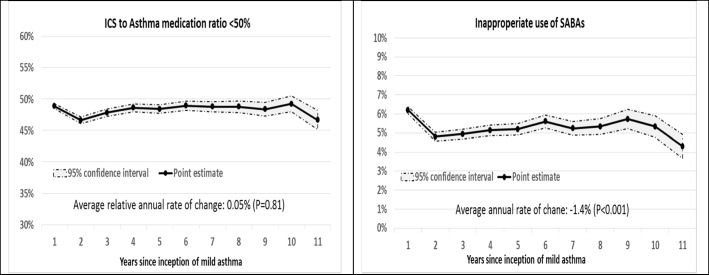
Trend of controller prescription to total medications (left panel) and inappropriate use of SABAs (right panel) over the time course of asthma

The trend in controller-to-total -medication ratio since diagnosis of asthma was not significant (*P* = 0.81). The proportion of individuals with inappropriate SABA prescription was 6.2% in the incident year of mild asthma. This ratio dropped sharply in the second year to 4.8% but had a slight increase to 5.4% during the next 10 years (Fig. 3 *– right panel*). The trend for this metric was consistent with 1.4% relative decrease per year, mainly due to the sharp decline form the first to the second year (*P* < 0.001).

### Factors associated with suboptimal controller and inappropriate SABA prescriptions

Results of the regression analyses are provided in Table 2. Individuals with higher socio-economic status as well as female patients had a lower likelihood of suboptimal controller prescriptions. Variables related to better quality of care, including the receipt of a pulmonary function test, non-urgent physician visits related to asthma (to both general practitioners and respiratory specialists but not to internal medicine specialists), and higher continuity of care had an inverse association with suboptimal controller prescription. Conversely, higher burden of comorbidity, higher severity of asthma, and history of asthma-related hospitalizations in the preceding period had positive association with suboptimal controller prescription. We observed generally similar patterns of association for inappropriate prescription of SABAs, aside from comorbidity and asthma-related hospitalizations, which were no longer statistically significant. In addition, asthma related general practitioner visits showed a positive association with inappropriate prescription of SABA.

**Table 2:** Factors associated with suboptimal controller use and inappropriate use of relievers

		Controller to total medications < 50%			Inappropriate SABA use		
Group	Variable	Odds Ratio	95% CI	*P* value	Odds Ratio	95% CI	*P* value
			(Lower, Upper)			(Lower, Upper)	
Socio-demographic	Sex (female = 1)	0.97	0.95–0.99	<.0003	0.78	0.76–0.81	<.0003
	Higher SES	0.97	0.97–0.98	<.0001	0.98	0.97–0.98	<.0001
	Year	0.99	0.98–1.00	<.0001	0.96	0.96–0.97	<.0001
	Age	0.81	0.81–0.82	<.0001	0.9	0.89–0.91	<.0001
Asthma treatment variables	Asthma-related hospitalization	1.63	1.51–1.76	<.0001	0.99	0.83–01.18	0.9222
	Asthma-related outpatient visit	0.95	0.94–0.96	<.0001	0.89	0.88–0.91	<.0001
	Oral corticosteroid	1.28	1.25–1.30	<.0001	0.4	0.38–0.41	<.0001
	Severity of Asthma	1.18	1.16–1.20	<.0001	3.6	3.52–3.69	<.0001
Comorbidity-related variables	Modified Charlson score	1.11	1.09–1.12	<.0001	0.98	0.96–1.00	0.1033
	None asthma related hospitalization	1.01	1.00–1.02	0.0009	1.01	1.00–1.03	0.0789
	None asthma related outpatient visit	1.00	1.00–1.00	<.0001	1.00	1.00–1.00	0.0137
Type & quality of care	Having received pulmonary function test	0.95	0.92–0.97	<.0003	0.87	0.82–0.92	<.0001
	Respirologist consultation	0.57	0.54–0.59	<.0001	0.54	049–0.60	<.0001
	Internal medicine consultation	0.98	0.93–1.03	0.5501	0.74	0.67–0.81	0.0014
	General Practitioner Asthma visit	0.67	0.66–0.68	<.0001	1.16	1.12–1.19	<.0001
	Continuity of care (COC)						
	COC = 0	–	–	–		–	–
	COC > 0 and COC < 50%	0.84	0.82–0.86	<.0001	0.76	0.74–0.79	<.0001
	COC > =50% and OC < 100%	0.86	0.84–0.88	<.0001	0.84	0.80–0.88	0.0014
	COC = 100%	0.86	0.83–0.83	<.0001	0.85	0.80–0.90	<.0001

*SABA* short-acting beta agonist, *SES* socio-economic status, *CI* confidence interval

## Discussion

We evaluated the indicators of balance between controller and reliever medications and their trends in 195,941 mild asthma patients during 14 years of longitudinal, population-based data. The controller/reliever balance was quantified as two metrics: suboptimal (< 0.50) prescription ratio of controllers to total asthma medications, and inappropriate prescription of SABAs according to previously published algorithms. Both metrics showed a decline over the period of 2000–2013. Over the entire follow up, the suboptimal prescription of controllers relatively dropped by almost 8% and inappropriate SABA prescription relatively declined by 20%. On the other hand, during the first eleven years of time course of mild asthma, the trends of suboptimal ICS prescription remained steady while inappropriate SABA prescription fell from the first to the second year but remained relatively constant afterwards. Both metrics were had the highest (worst) values for patients in their twenties. We also evaluated the association between such metrics and multiple factors related to patient or disease characteristics and type and quality of care. Among the studied factors, the indices of type and quality of care, namely continuity of care and respirologist consultation, had the strongest associations with improvement in controller/reliever balance. As well, higher socio-economic status and female sex were associated with more appropriate and balanced prescriptions of asthma medications.

Previous studies have evaluated the trends in asthma medications [24–27]. Highasi et al. used data from two national surveys in the United States to document trends of asthma medications between 1997 and 2008 [24]. Their results indicated declines in the prescriptions of SABAs and increase in ICS prescriptions. They also indicated an increase in the ratio of controller to total asthma medications from 0.5 in 1997 to a peak of 0.7 in 2004. Similarly, Johnson et al. used data from a European prospective cohort study with an average follow-up of 8.7 years [25]. In the sample with asthma (*n*  =  423), the prescriptions for ICS increased by 12.2% over a 10-year period. Despite this, only 17.2% were using ICS on a daily basis during follow-up.

To the best of our knowledge there has not been any studies on medication balance in mild asthma, which constitutes the majority of asthma patients. In addition evaluating trends over age and over the time course of the disease are novel features of our design. We showed a decline in the inappropriate prescription of controllers and relievers, especially for the latter, between the first and second year. This can be due to higher prescription of reliever medications and avoidance of ICS in early stage of asthma [24]. We also showed that controller/reliever balance was worse in the patients in their twenties. This might be explained by lower adherence to controller therapies in adolescents and young adults, as observed elsewhere [28]; alternatively it might indicate a failure of proper transfer of care from the pediatrician to the adult care provider.

Several factors can affect the way asthma medications are prescribed by care providers and used by patients. Within limitations of administrative health data, we evaluated multiple factors ranging from socio-demographic variables to disease characteristics, type and quality of care, and comorbidity. Our results are consistent with previous studies with regard to higher socio-economic status and female sex being associated with more balanced use of asthma medications [29–31]. Other observed associations appear to be reported for the first time in this study. For instance, continuity of care was associated with better asthma medication use. Interestingly, general practitioner visits were associated with an increased risk of inappropriate SABA prescriptions but a decreased risk of low controller-to-total-asthma-medication ratio. Respirologist consultation was associated with improvement in both indices. This discrepancy might be due to the differences among the guidelines available to each group, or their adherence in following best practice recommendations. Interestingly, asthma-related hospitalization in the previous year increased the risk of suboptimal prescription of controller, which can be a concerning factor.

A notable association was observed between comorbidity and suboptimal controller prescription. This association was our a priori hypothesis, based on the observed association between age and suboptimal asthma medication use in the general population in our previous work [26]. We hypothesized that a factor that might increase suboptimal use of asthma medications is the higher burden of comorbidity in the older age groups, which might direct the patient’s and care provider’s attention away from the proper management of asthma. The association with comorbidity was only statistically significant for controller-to-total-asthma medication ratio but not for inappropriate SABA prescription. In an exploratory analysis, when we divided the Charlson comorbidity index into its constituents, almost all diseases remained positively associated with suboptimal controller prescription (Additional file 2: Table S2).

Using a large population-based sample of entire population of a well-defined geographical region with a long follow-up period is the key strength of this study. Covering the entire population of a jurisdiction with a public healthcare system, the study sample is nearly free of the selection bias that affects clinical studies or claims-based records from third-party insurers. This, combined with the long follow-up time, allowed us to estimate trends over different time axes with a low level of uncertainty. The large sample size enabled us to robustly evaluate the association between multiple factors and controller/reliever balance. There are also some limitations in our study. While validated specifically for the Canadian context and used in several previous studies [32], the classification of asthma based on severity relies on health services use and medication records; as such it can be affected by many factors such as lack of adherence to medications. Second, filling prescriptions is not equal to medication intake. However, we deem it unlikely that any discrepancy between filled prescriptions and actual intake would substantially change over time; therefore, such discrepancy is unlikely to threaten the validity of the analyses of trends. In addition, we did not have information about important factors such as patients’ smoking status and degree of airway limitation. Further, classification of patient-years into asthma severity categories was independent of the previous history of asthma. As such, some patient-years that were classified as mild asthma after a severe asthma episode were included in the analytical dataset. Given the objective of this study, the inclusion of such periods is justified, but asthma patients with a past history of severe episodes might be treated differently. In a secondary analysis (results not shown) when such patient-years were removed, the overall findings stayed the same.

## Conclusion

In summary, suboptimal prescription of asthma medications are prevalent in mild asthma. Although the declining trends over time are encouraging, given the sheer size of mild asthma population, still unacceptably large number of patients are exposed to inappropriate doses of reliever medications. For example, our results indicate that in 2013, out of 45,139 non-dormant mild asthma patients in BC, 22,153 individuals were exposed to low doses of controller therapies in relation with total asthma medications, and 3056 patients had indicators of inappropriate reliever medication prescription. Recent evidence suggests the benefit of earlier use of controller therapy in mild asthma patients [8, 9] and our results indicate that there is substantial room for such evidence to be translated into practice towards improvement of patient outcomes in mild asthma.

## Additional files

Additional file 1: **Table S1.** List of asthma related medication. (DOCX 49 kb)

Additional file 2: **Table S2.**  Odd ratios of association between comorbid conditions on ICS to total medications < 50%. (DOCX 15 kb)

## Abbreviations

BC: British Columbia
GLM: Generalized Liner Models
ICD: International Classification of Disease
ICS: Inhaled Corticosteroids
P: *P*-values
SABA: Short-Acting Beta-Agonists
