# Supplementary material for: Extent, trends, and determinants of controller/reliever balance in mild asthma: a 14-year population-based study
Source: Respir Res. 2019 Feb 28;20:44. doi: 10.1186/s12931-019-1007-0 (PMC6394061; doi:10.1186/s12931-019-1007-0)
Supplement: Supplementary file 1 — Table S1. List of asthma related medication. (DOCX 49 kb) [file 12931_2019_1007_MOESM1_ESM.docx]

Table S1: List of asthma related medication

| # | Brand Name | # | Brand Name | # | Brand Name |
| --- | --- | --- | --- | --- | --- |
| **1** | ALUPENT LIQ 50MG/ML | **151** | A-HYDROCORT INJ 1000MG/8ML | **301** | SOLU-MEDROL 125 MG STERILE POWDER |
| **2** | ALUPENT TAB 20MG | **152** | A-HYDROCORT INJ 100MG/2ML | **302** | SOLU-MEDROL 40 MG STERILE POWDER |
| **3** | AMINOPHYLLINE TAB 0.1GM | **153** | EPHEDRINE TAB 15MG | **303** | NOVO-KETOTIFEN TAB 1MG |
| **4** | ARISTOCORT TAB 2MG | **154** | EPHEDRINE TAB 30MG | **304** | FORADIL DRY POWDER CAPSULES FOR INH |
| **5** | ARISTOCORT TAB 4MG | **155** | BECLOVENT AEM 50MCG/AEM | **305** | SEREVENT DISKUS (50MCG/DOSE) |
| **6** | CORTONE SUS 50MG/ML | **156** | VENTOLIN IM INJ 0.5MG/ML | **306** | PMS-IPRATROPIUM |
| **7** | CORTONE TAB 5MG | **157** | VENTOLIN IV BOLUS INJ 0.05MG/ML | **307** | PMS-IPRATROPIUM (20 ML BOT |
| **8** | CORTONE TAB 25MG | **158** | VENTOLIN NEBULES P.F. SOL 2.5MG/2.5 | **308** | PMS-IPRATROPIUM (1ML UNIT DOS |
| **9** | DECADRON TAB 0.5MG | **159** | BECLOFORTE 250MCG/AEM | **309** | PMS-IPRATROPIUM (2ML UNIT DO |
| **10** | NOVO-PREDNISONE 5MG | **160** | ISUPREL LIQ INH 0.5% | **310** | GEN-CROMOLYN NASAL SPRAY |
| **11** | CELESTONE SOLUSPAN INJECTABLE | **161** | SLO-BID 100 | **311** | APO-CROMOLYN NASAL SPRAY |
| **12** | CELESTONE TAB 0.5MG | **162** | SLO-BID 200 | **312** | ASMAVENT NEBULES P.F. |
| **13** | SOLU-CORTEF 100 MG ACT-O-VIAL | **163** | SLO-BID 300 | **313** | APO-CROMOLYN STERULES |
| **14** | SOLU-CORTEF 250 MG ACT-O-VIAL | **164** | SLO-BID 50 | **314** | APO-SALVENT STERULES |
| **15** | SOLU-CORTEF 500 MG ACT-O-VIAL | **165** | THEO-SR 300 SRT 300MG | **315** | APO-IPRAVENT STERULES |
| **16** | SOLU-CORTEF 1 GM ACT-O-VIAL | **166** | MYLAN-SALBUTAMOL STERINEBS P.F. | **316** | NU-CROMOLYN PLASTIC AMPULES |
| **17** | SOLUMEDROL INJ 40MG STERILE MIXOVIA | **167** | VAPONEFRIN | **317** | APO-SALVENT STERULES |
| **18** | SOLUMEDROL INJ 125MG STERILE MIXOVI | **168** | ISUPREL MISTOMETER 125MCG/AEM | **318** | PMS-KETOTIFEN |
| **19** | SOLU-MEDROL 500 MG STERILE POWDER | **169** | VENTOLIN TAB 4MG | **319** | PMS-KETOTIFEN |
| **20** | DEPO-MEDROL 40 MG/ML | **170** | DEPO-MEDROL (WITH PRESERVATIVE) 20 | **320** | NU-SALBUTAMOL PLASTIC AMPULES 1 MG/ML |
| **21** | DEPO-MEDROL 80 MG/ML | **171** | DEPO-MEDROL (WITH PRESERVATIVE) 40 | **321** | NU-SALBUTAMOL PLASTIC AMPULES 2 MG/ML |
| **22** | CORTEF TABLETS 10MG | **172** | DEPO-MEDROL (WITH PRESERVATIVE) 80 | **322** | NU-IPRATROPIUM PLASTIC AMPULES 250 MCG |
| **23** | CORTEF TABLETS 20 MG | **173** | VENTOLIN ROTACAPS 400MCG | **323** | METHYLPREDNISOLONE SODIUM SUCCINATE |
| **24** | MEDROL 4 MG | **174** | VENTOLIN ROTACAPS 200MCG | **324** | METHYLPREDNISOLONE SODIUM SUCCINATE |
| **25** | MEDROL 16 MG | **175** | VENTOLIN NEBULES PF SOL 5MG/2.5ML | **325** | METHYLPREDNISOLONE SODIUM SUCCINATE |
| **26** | SOLU-MEDROL 1 G STERILE POWDER | **176** | PMS DEXAMETHASONE ELIXIR 0.5MG/5ML | **326** | AIROMIR |
| **27** | BETNESOL TAB 0.5MG | **177** | VENTOLIN RESPIRATOR SOLUTION 5MG/ML | **327** | "METHYLPREDNISOLONE SODIUM SUCCINATE F |
| **28** | EPHEDRINE SULFATE INJECTION | **178** | BECLOVENT ROTACAPS 100MCG | **328** | METHYLPREDNISOLONE SODIUM SUCCINATE |
| **29** | THEOPHYLLINE ELIXIR | **179** | BECLOVENT ROTACAPS 200MCG | **329** | MYLAN-SALBUTAMOL RESPIRATOR SOLUTIO |
| **30** | PREDNISONE TAB 5MG | **180** | RHINARIS-CS ANTI-ALLERGIC NASAL MIST | **330** | ACCOLATE TAB 20 MG |
| **31** | CELESTONE REPETABS 1MG | **181** | ATROVENT UDV SOL INH 250MCG/ML | **331** | EPHEDRINE HCL |
| **32** | AMINOPHYLLINE TAB 100MG | **182** | PMS DEXAMETHASONE TAB 4MG | **332** | APO-ORCIPRENALINE SYRUP |
| **33** | DELTASONE 5MG | **183** | PMS DEXAMETHASONE TAB 0.75MG USP | **333** | PHL-SALBUTAMOL RESPIRATOR SOLU |
| **34** | DECADRON PHOSPHATE INJ 4MG/ML | **184** | PMS DEXAMETHASONE TAB 0.5MG USP | **334** | PHL-SALBUTAMOL RESPIRATOR SOLU |
| **35** | NOVO-PREDNISONE TAB 50MG | **185** | THEOLAIR LIQ 80MG/15ML | **335** | PHL-SALBUTAMOL RESPIRATOR SOLU |
| **36** | ALUPENT SYR 10MG/5.0ML | **186** | THEOLAIR TAB 250MG | **336** | PHL-IPRATROPIUM |
| **37** | DELTASONE 50MG | **187** | THEOLAIR TAB 125MG | **337** | PHL-IPRATROPIUM - 20ML |
| **38** | ALUPENT AER | **188** | THEOLAIR SR TAB 200MG | **338** | PHL-DEXAMETHASONE |
| **39** | THEOPHYLLINE 80MG/15ML | **189** | THEOLAIR SR TAB 250MG | **339** | PHL-DEXAMETHASONE |
| **40** | INTAL SPINCAPS 20MG | **190** | THEOLAIR SR TAB 300MG | **340** | PHL-DEXAMETHASONE |
| **41** | WINPRED TAB 1MG | **191** | THEOLAIR SR TAB 500MG | **341** | EPHEDRA |
| **42** | WINPRED TAB 5MG | **192** | DEXAMETHASONE SODIUM PHOS INJ 4MG/M | **342** | PHL-IPRATROPIUM - 1ML POLYNEB |
| **43** | CORTISONE ACETATE TAB 25MG | **193** | PULMICORT NEBUAMP 0.25 MG/ML | **343** | PHL-IPRATROPIUM - 2ML POLYNEB |
| **44** | DEXASONE 0.75MG | **194** | PULMICORT NEBUAMP 0.5 MG/ML | **344** | OXEZE TURBUHALER 12MCG/AEM |
| **45** | DEXASONE 0.5MG | **195** | RATIO-SALBUTAMOL | **345** | OXEZE TURBUHALER 6 MCG/AEM |
| **46** | THEOPHYLLINE ROUGIER ELIXIR | **196** | EPHEDRINE HYDROCHLORIDE 25MG T | **346** | FLOVENT DISKUS |
| **47** | APO PREDNISONE TAB 5MG | **197** | UNIPHYL | **347** | FLOVENT DISKUS |
| **48** | RATIO-THEO-BRONC | **198** | UNIPHYL | **348** | FLOVENT DISKUS |
| **49** | I D M EXPECTORANT TAB | **199** | PHYLLOCONTIN | **349** | FLOVENT DISKUS |
| **50** | ROUPHYLLINE TAB 200MG | **200** | PHYLLOCONTIN-350 | **350** | BETAJECT |
| **51** | DECADRON TAB 4MG | **201** | BRONKAID MISTOMETER AEM 0.5% | **351** | SINGULAIR |
| **52** | IDM TAB | **202** | ISUPREL LIQ 0.5% | **352** | SINGULAIR |
| **53** | VANCERIL AEM 50MCG | **203** | ISUPREL MISTOMETER AEM 0.25% | **353** | MYLAN-IPRATROPIUM SOLUTION |
| **54** | ROUPHYLLINE SYR 10MG/ML | **204** | LIVOSTIN SUS NAS 0.5MG/ML | **354** | RATIO-SALBUTAMOL |
| **55** | FORMULA C34 TAB .13MG | **205** | VENTOLIN NEBULES P.F. LIQ INH 1.25M | **355** | RATIO-SALBUTAMOL |
| **56** | APO OXTRIPHYLLINE TAB 100MG | **206** | ATROVENT UDV 125MCG/ML | **356** | DOM-DEXAMETHASONE TABLET 4MG |
| **57** | APO OXTRIPHYLLINE TAB 200MG | **207** | VENTOLIN ORAL LIQUID 0.4MG/ML | **357** | PMS-IPRATROPIUM |
| **58** | ROUPHYLLINE TAB 100MG | **208** | PMS-SODIUM CROMOGLYCATE | **358** | RATIO-IPRATROPIUM |
| **59** | NOVO-TRIPHYL TAB 100MG | **209** | APO-SALVENT 5MG/ML | **359** | DOM-IPRATROPIUM |
| **60** | NOVO-TRIPHYL TAB 200MG | **210** | ASMAVENT RESPIRATOR SOLUTION INH 5MG/ | **360** | RATIO-DEXAMETHASONE TABLETS, USP |
| **61** | THEO-DUR 100 MG | **211** | NOVO-CROMOLYN NEBULIZER SOLUTION 1% | **361** | "RATIO-DEXAMETHASONE TABLETS |
| **62** | THEO-DUR 200 MG | **212** | BETNESOL TABLETS | **362** | RATIO-DEXAMETHASONE TABLETS, USP |
| **63** | THEO-DUR 300 MG | **213** | VENTOLIN INJECTION LIQ IV 5MG/5ML | **363** | ADVAIR 100 DISKUS |
| **64** | PULMOPHYLLINE ELX | **214** | SOLU-MEDROL - (ACT-O-VIAL 1G STERIL | **364** | ADVAIR 250 DISKUS |
| **65** | DYSNE INHAL | **215** | SOLU-MEDROL - (ACT-O-VIAL 500MG STE | **365** | ADVAIR 500 DISKUS |
| **66** | CHOLEDYL ELIXIR 100MG/5ML | **216** | SOLU-MEDROL - (ACT-O-VIAL 40MG STER | **366** | METHYLPREDNISOLONE SODIUM SUCCINATE FO |
| **67** | CHOLEDYL EXPECTORANT ELIXIR | **217** | SOLU-MEDROL - (ACT-O-VIAL 125MG STE | **367** | VENTOLIN HFA |
| **68** | CHOLEDYL PEDIATRIC SYR 50MG/5ML | **218** | PMS-SALBUTAMOL | **368** | QVAR 50 MCG |
| **69** | CHOLEDYL TAB 200MG | **219** | BECLOMETHASONE DIPROPIONATE ORAL INHALER | **369** | QVAR 100 MCG |
| **70** | THEOPHYLLINE ELIXIR | **220** | MED SALBUTAMOL INHALATION SOLUTION 1MG/ML | **370** | FORMULA T.L. |
| **71** | DEXASONE 4MG | **221** | PMS-SALBUTAMOL ORAL LIQUID | **371** | EPHEDRINE SULFATE INJECTION |
| **72** | AMINOPHYLLINE INJECTION 25MG/M | **222** | RATIO-IPRATROPIUM INHALATION SOLUTION | **372** | VENTOLIN DISKUS |
| **73** | AMINOPHYLLINE INJECTION 50MG/ML | **223** | RATIO-IPRATROPIUM UDV | **373** | EPHEDRINE 8MG |
| **74** | CHOLEDYL SA TABLETS 400MG | **224** | RATIO-IPRATROPIUM UDV | **374** | SINGULAIR |
| **75** | PREDNISONE TAB 5MG | **225** | EPHEDRINE 15 MG TAB | **375** | APO-IPRAVENT STERULES |
| **76** | APO OXTRIPHYLLINE TAB 300MG | **226** | EPHEDRINE 30 MG TABLETS | **376** | APO-SALVENT STERULES |
| **77** | S2 | **227** | APO-IPRAVENT SOLUTION - INH 250MCG/ | **377** | FLOVENT HFA |
| **78** | THEOPHYLLINE ELX | **228** | #N/A | **378** | FLOVENT HFA |
| **79** | INTAL NEBULIZER 1% | **229** | #N/A | **379** | FLOVENT HFA |
| **80** | CHOLEDYL SA TABLETS 600MG | **230** | SEREVENT- AEM 25MCG/AEM | **380** | RATIO-SALBUTAMOL HFA |
| **81** | BRONCHYL SYR | **231** | SEREVENT-PWR 50MCG/BLISTER PACK | **381** | ADVAIR 125 |
| **82** | APO PREDNISONE TAB 50MG | **232** | APO-SALVENT TABLETS - 2MG | **382** | ADVAIR 250 |
| **83** | INTAL INHALER | **233** | APO-SALVENT TABLETS - 4MG | **383** | SYMBICORT 100 TURBUHALER |
| **84** | QUIBRON T SR TAB 300MG | **234** | SALBUTAMOL INHALATION SOLUTION | **384** | SYMBICORT 200 TURBUHALER |
| **85** | NOVO-TRIPHYL TAB 300MG | **235** | DUOVENT UDV | **385** | METHYLPREDNISOLONE ACETATE INJECTAB |
| **86** | PMS-THEOPHYLLINE ELIXIR | **236** | RATIO-ORCIPRENALINE SYRUP 2MG/ML | **386** | METHYLPREDNISOLONE ACETATE INJECTAB |
| **87** | ATROVENT AEM 28.6MG/100GM | **237** | SANDOZ SALBUTAMOL | **387** | METHYLPREDNISOLONE ACETATE INJECTAB |
| **88** | ZADITEN | **238** | ATROVENT NASAL SPRAY - 21MCG/AEM | **388** | METHYLPREDNISOLONE ACETATE INJECTAB |
| **89** | AMINOPHYLLINE INJ 50MG/ML | **239** | ATROVENT NASAL SPRAY - 42MCG/AEM | **389** | APO-SALVENT CFC FREE |
| **90** | AMINOPHYLLINE INJ 25MG/ML | **240** | SALBU-2 - TAB 2MG | **390** | APO-IPRAVENT NASAL SPRAY |
| **91** | OXTRIPHYLLINE TAB 300MG | **241** | SALBU-4 - TAB 4MG | **391** | APO-IPRAVENT NASAL SPRAY |
| **92** | OXTRIPHYLLINE TAB 200MG | **242** | NU-SALBUTAMOL - TAB 2MG | **392** | SPIRIVA |
| **93** | APO PREDNISONE TAB 1MG USP | **243** | NU-SALBUTAMOL - TAB 4MG | **393** | ATROVENT HFA |
| **94** | THEOCHRON SRT 300MG | **244** | MYLAN-SALBUTAMOL STERINEBS P.F. | **394** | SINGULAIR |
| **95** | ZADITEN | **245** | FLOVENT INHALERS-AEM INH-ORL 25MCG/ | **395** | APO-DEXAMETHASONE |
| **96** | RYNACROM SOLUTION 2% | **246** | FLOVENT INHALERS-AEM INH-ORL 50MCG/ | **396** | BCI SALBUTAMOL INHALATION SOLU |
| **97** | PREDNISONE 50 TAB 50MG | **247** | FLOVENT INHALERS-AEM INH-ORL125MCG/ | **397** | PHL-DEXAMETHASONE |
| **98** | ALTI-PREDNISONE TAB 5MG | **248** | FLOVENT INHALERS-AEM INH-ORL250MCG/ | **398** | PHL-DEXAMETHASONE |
| **99** | NOVO-SALMOL TAB 2MG | **249** | NOVO-KETOTIFEN - SYR 1MG/5ML | **399** | XOLAIR |
| **100** | NOVO-SALMOL TAB 4MG | **250** | TANTA ORCIPRENALINE SYRUP - SY | **400** | APO-DEXAMETHASONE |
| **101** | ELIXIR DE THEOPHYLLINE | **251** | ARISTOCORT-TAB 2MG | **401** | PHL-SALBUTAMOL ORAL LIQUID |
| **102** | THEOCHRON SRT 100MG | **252** | ARISTOCORT - TAB 4MG | **402** | PMS-DEXAMETHASONE |
| **103** | THEOCHRON SRT 200MG | **253** | DEXAMETHASONE-OMEGA | **403** | ALVESCO |
| **104** | INTAL SYNCRONER | **254** | DEXAMETHASONE-OMEGA | **404** | ALVESCO |
| **105** | THEOPHYLLINE 0.8MG/ML IN 5% DE | **255** | PMS-SALBUTAMOL | **405** | OMNARIS |
| **106** | DEXAMETHASONE SODIUM PHOSPHATE INJ | **256** | PMS-SALBUTAMOL | **406** | PRO-DEXAMETHASONE - 4 |
| **107** | APO-THEO-LA SRT 100MG | **257** | PMS-SALBUTAMOL | **407** | NOVO-SALBUTAMOL HFA |
| **108** | APO-THEO-LA SRT 200MG | **258** | NOVO-IPRAMIDE - LIQ 0.25MG/ML | **408** | Nucala |
| **109** | APO-THEO-LA SRT 300MG | **259** | SEREVENT (25MCG/ACTUATION) | **409** | Cinqair |
| **110** | DEXAMETHASONE SOD PHOS INJ 4MG/ML | **260** | VENTOLIN ROTACAPS | **410** | Breo 100 mcg |
| **111** | PULMO SEPTOL SIROP | **261** | VENTOLIN ROTACAPS | **411** | Breo 200 mcg |
| **112** | THEO-DUR 450 MG | **262** | VENTOLIN ORAL LIQUID | **412** | SPIRIVA RESPIMAT 2.5mg/act |
| **113** | ATROVENT | **263** | VENTOLIN NEBULES P.F.- 1.25MG/2.5ML | **413** | Asmanex 200mcg |
| **114** | HEXADROL PHOSPHATE INJ 4MG/ML | **264** | VENTOLIN NEBULES P.F. - 2.5MG/2.5ML | **414** | Asmanex 400mcg |
| **115** | HEXADROL PHOSPHATE INJ 10MG/ML | **265** | VENTOLIN NEBULES P.F. - 5MG/2.5ML | **415** | Asmanex 100mcg |
| **116** | PMS-DEXAMETHASONE SOD PHOSPHATE INJ 4M | **266** | VENTOLIN I.V. BOLUS INJECTION 0.25M | **416** | Zenhale 50/5mcg |
| **117** | PMS-DEXAMETHASONE SOD PHOSPHATE INJ | **267** | VENTOLIN I.M. INJECTION 0.5MG SALBU | **417** | Zenhale 100/5mcg |
| **118** | BRICANYL TURBUHALER 0.5 MG/AEM | **268** | VENTOLIN I.V. INFUSION SOLUTIO | **418** | Zenhale 200/5mcg |
| **119** | APO-SALVENT AEM 100MCG | **269** | VENTOLIN INHALER | **419** | Arnuity 100 mcg |
| **120** | PMS OXTRIPHYLLINE SYR PEDIATRIC 10MG/M | **270** | VENTOLIN RESPIRATOR SOLUTION | **420** | Arnuity 200 mcg |
| **121** | PMS-OXTRIPHYLLINE ELIXIR | **271** | FLOVENT INHALERS - AEM INH-ORL 25MC | **421** | COMBIVENT INHALATION AEROSOL |
| **122** | SABULIN INHALER 100MCG/METERED | **272** | FLOVENT INHALERS - AEM INH-ORL 50MC | **422** | COMBIVENT RESPIMAT |
| **123** | ATROVENT NASAL AEROSOL 20MCG/AEM | **273** | FLOVENT INHALERS - AEM INH-ORL 125M | **423** | COMBIVENT UDV |
| **124** | BECLODISK PWR 100MCG/BLISTER | **274** | FLOVENT INHALERS - AEM INH-ORL 250M | **424** | SYMBICORT FORTE TURBUHALER |
| **125** | BECLODISK PWR 200MCG/BLISTER | **275** | BECLODISK - PWR INH 100MCG/BLISTE | **425** | Bricanyl |
| **126** | THEOPHYLLINE 0.8MG AND 5% DEXT | **276** | BECLODISK - PWR INH 200MCG/BLISTER | **426** | Bricanyl |
| **127** | THEOPHYLLINE 1.6MG AND 5% DEXT | **277** | SEREVENT DISKHALER DISK (50MCG/DOSE | **427** | Aeromony Respiclick |
| **128** | THEOPHYLLINE 4MG AND 5% DEXTRO | **278** | VENTODISK 200MCG | **428** | Aeromony Respiclick |
| **129** | VENTODISK PWR 400MCG/BLISTER | **279** | VENTODISK 400MCG | **429** | Aeromony Respiclick |
| **130** | VENTODISK PWR 200MCG/BLISTER | **280** | BECLOVENT ROTACAPS - INH 100MCG/C | **430** | Anoro |
| **131** | PULMICORT TURBUHALER 200 MCG/DOSE | **281** | BECLOVENT ROTACAPS - INH 200MCG/C | **431** | Incruse |
| **132** | PULMICORT TURBUHALER 400 MCG/DOSE | **282** | BECLOFORTE INHALER - AEM INH 250MCG | **432** | Seebri |
| **133** | RATIO-SALBUTAMOL | **283** | SALBUTAMOL NEBUAMP 0.05% | **433** | ULTIBRO BREEZHALER |
| **134** | PULMICORT TURBUHALER 100 MCG/DOSE | **284** | SALBUTAMOL NEBUAMP 0.1% | **434** | ONBREZ BREEZHALER |
| **135** | RATIO-SALBUTAMOL | **285** | SALBUTAMOL NEBUAMP 0.2% | **435** | INSPIOLTO RESPIMAT |
| **136** | VENTOLIN INHALER 100MCG/AEM | **286** | MYLAN-IPRATROPIUM STERINEBS | **436** | STRIVERDI RESPIMAT |
| **137** | JAA PREDNISONE TAB 1MG USP | **287** | BECLOVENT - AEM 50MCG/AEM | **437** | TUDORZA GENUAIR |
| **138** | JAA PREDNISONE TAB 50MG USP | **288** | DOM-SALBUTAMOL RESPIRATOR SOLU | **438** | DUAKLIR GENUAIR |
| **139** | JAA PREDNISONE TAB 5MG USP | **289** | NU-KETOTIFEN SYRUP - 1MG/5ML |  |  |
| **140** | JAA AMINOPHYLLINE TAB 100MG | **290** | GEN-CROMOGLYCATE STERINEBS - LIQ INH 1 |  |  |
| **141** | ALTI-BECLOMETHASONE DIPROPIONATE INHAL | **291** | EPHEDRINE HCL - TAB 25MG |  |  |
| **142** | HYDROCORTISONE SOD SUCCINATE INJ 10 | **292** | APO-KETOTIFEN - SYR 1MG/5ML |  |  |
| **143** | HYDROCORTISONE SOD SUCCINATE INJ 25 | **293** | PULMICORT NEBUAMP 0.125 MG/ML |  |  |
| **144** | NOVO-SALMOL INHALER 100MCG/AEM | **294** | #N/A |  |  |
| **145** | DEXAMETHASONE SOD PHOSPH INJ 10MG/ML U | **295** | EPHEDRA |  |  |
| **146** | EPHEDRINE SULFATE INJECTION US | **296** | EPHEDRINE HYDROCHLORIDE TABLET |  |  |
| **147** | HYDROCORTISONE SODIUM SUCCIN INJ 50 | **297** | ORCIPREN |  |  |
| **148** | HYDROCORTISONE SODIUM SUCCINATE INJ | **298** | NOVO-THEOPHYL SR |  |  |
| **149** | A-HYDROCORT INJ 250MG/2ML | **299** | NOVO-THEOPHYL SR |  |  |
| **150** | A-HYDROCORT INJ 500MG/4ML | **300** | NOVO-THEOPHYL SR |  |  |

An example of dose-equivalence calculations for inhaled corticosteroids

Consider a patient who, during the one-year period of interest, is prescribed 3 canisters (120 puffs each) of fluticasone propionate 250mcg/dose for twice daily use, as well as 1 canister of salbutamol (Ventolin) with 400mcg of salbutamol for an average of twice weekly use (200 puffs each). Note that such information is available within filled prescription records, in terms of total dispensed doses and days of supply. The patient has not filled any other asthma-related medications.

The dose-equivalency for fluticasone is 2 (every 1mcg of fluticasone is equal to 2mcg of beclomethasone in potency), and the reference is beclomethasone dipropionate (250mcg, 120 puffs per dose). Therefore, the total beclomethasone-equivalent daily ICS dose for this patient will be

Total ICS dose during the year= 3 * 120 * 2 = 720

As for salbutamol, its dose equivalence is 2; because the reference category is salbutamol 200mcg/puff. The calculation of total SABA use is

Total SABA use during the year= 1* 200 * 2 = 400

As such, the ratio of controller to total medication for this patient is

ratio=ICS/(ICS+SABA)=720/(720+400)=64.2%

For this patient, this pattern of medication prescription is considered ‘appropriate’ given that the above-mentioned ratio is above 50%
