# Supplementary material for: Extent, trends, and determinants of controller/reliever balance in mild asthma: a 14-year population-based study
Source: Respir Res. 2019 Feb 28;20:44. doi: 10.1186/s12931-019-1007-0 (PMC6394061; doi:10.1186/s12931-019-1007-0)
Supplement: Supplementary file 2 — Table S2. Odd ratios of association between comorbid conditions on ICS to total medications < 50%. (DOCX 15 kb) [file 12931_2019_1007_MOESM2_ESM.docx]

Table S2: Odd ratios of associated comorbid conditions on ICS to total medications <50%

|  | Odds ratio | Lower limit | Upper limit |
| --- | --- | --- | --- |
| Myocardial Infarction | 1.282 | 1.107 | 1.485 |
| Congestive Heart Failure | 1.101 | 0.994 | 1.219 |
| Peripheral Vascular Disease | 1.219 | 1.106 | 1.343 |
| Cerebrovascular Disease | 1.222 | 1.102 | 1.355 |
| Dementia | 1.097 | 0.944 | 1.273 |
| Connective Tissue-Rheumatic Disease | 1.665 | 1.567 | 1.769 |
| Peptic Ulcer Disease | 1.130 | 1.055 | 1.210 |
| Mild Liver Disease | 1.135 | 1.070 | 1.205 |
| Diabetes without complications | 1.022 | 0.984 | 1.062 |
| Diabetes with complications | 1.201 | 1.110 | 1.300 |
| Paraplegia and Hemiplegia | 1.113 | 1.019 | 1.216 |
| Renal Disease | 1.278 | 1.216 | 1.342 |
| Cancer | 1.119 | 1.086 | 1.154 |
| Moderate or Severe Liver Disease | 1.068 | 1.013 | 1.125 |
| Metastatic Carcinoma | 1.271 | 1.215 | 1.328 |
| AIDS/HIV | 1.072 | 1.044 | 1.101 |
